# Comparison of CT-guided intracystic lidocaine pre-injection versus conventional US-guided single-session ethanol sclerotherapy for simple hepatic cysts

**DOI:** 10.1038/s41598-025-98140-7

**Published:** 2025-04-17

**Authors:** Hao Zhang, Chi Zhang, Ye Liu, Lin Li, Jian-xue Duan, Jian-ning Zhao

**Affiliations:** 1 Department of Radiology, Dianjiang People’s Hospital of Chongqing, 116 North Street, Guixi Street, Dianjiang County, Chongqing, 408300 People’s Republic of China; 2 Class 21, Grade 2025, Chongqing Yangjiaping Middle School, Chongqing, 400050 People’s Republic of China; 3 Department of Pharmacy, Dianjiang People’s Hospital of Chongqing, 116 North Street, Guixi Street, Dianjiang County, Chongqing, 408300 People’s Republic of China; 4 Department of Hepatobiliary Surgery, Dianjiang People’s Hospital of Chongqing, Chongqing, 408300 People’s Republic of China; 5 Department of Radiology, Chongqing Traditional Chinese Medicine Hospital, No. 6, Panxi Seven Branch Road, Jiangbei District, Chongqing, 400013 People’s Republic of China

**Keywords:** Simple hepatic cysts, Single-session, Ethanol sclerotherapy, Ablation, Pain, Tomography, Randomized controlled trials, Biliary tract disease, Radiography

## Abstract

This study aimed to compare a modified CT-guided technique incorporating intracystic lidocaine for pain control versus a conventional ultrasound-guided method for single-session ethanol sclerotherapy of simple hepatic cysts (SHCs), to seek refinements in technical strategies. 113 patients with SHCs underwent ethanol sclerotherapy between January 2019 and June 2023 at two centers. Center A utilized a modified CT-guided technique with intracystic lidocaine pre-injection into the cyst. Center B employed a conventional ultrasound-guided method with pigtail catheter drainage. Primary endpoints included pain during ethanol injection, procedural duration, and safety, while secondary endpoints assessed cyst volume reduction and symptom relief. 99 eligible patients (Center A/B, n = 43/56) were included in the analysis. The modified technique at Center A resulted in significantly lower median VAS scores (1.2 ± 1.2) compared to Center B (5.1 ± 1.1; *p* < 0.01) during ethanol injection. Ethanol exposure and procedural durations were also shorter at Center A (11.2 ± 1.6 min and 29.9 ± 4.3 min) than at Center B (20.5 ± 3.4 min and 40.6 ± 4.2 min; *p* < 0.01). No complications were observed at Center A. Both centers achieved similar success rates in reducing cyst size and relieving symptoms at the 6-month follow-up. The modified CT-guided percutaneous single-session ethanol sclerotherapy technique for SHCs significantly reduces pain and procedural duration compared to the conventional method, with similar efficacy and safety profiles.

## Introduction

Simple hepatic cysts (SHCs) reportedly originate from a congenital biliary malformation, but they do not exhibit biliary system communication. The wall is lined with cubic biliary epithelium, and the cavity is filled with serous fluid. Over time, some cysts continuously grow with fluid secreted by epithelial cells^1^, causing symptomatic or complicated disease or patient anxiety, impairing quality of life. Therefore, SHCs can be treated through various approaches, including laparoscopic deroofing and percutaneous interventions. Among percutaneous techniques^2^, ethanol aspiration sclerotherapy has been a routine procedure for more than two decades because it is simple, effective, safe, and inexpensive^3^. However, pain induced by ethanol injection remains the most common adverse event, often forcing operators to inject smaller ethanol volumes or even suspend or discontinue the procedure in severe cases^4–6^. Alternatively, the procedure may be performed with conscious sedation, analgesics, or under general anesthesia^7,8^. Addressing this issue is crucial for improving patient compliance and procedural outcomes.

Various technical approaches for ethanol sclerotherapy have evolved over time, with the most common being ultrasound or CT-guided drainage using either pigtail catheters or coaxial needles. At the local medical center, the procedure was modified in specific technical aspects, achieving the desired effect: pain was controlled, and procedural efficiency was improved. An appropriate comparative assessment of these modifications against conventional techniques was needed to validate these promising preliminary observations. Therefore, this study aims to compare two distinct technical approaches for single-session SHC sclerotherapy: a modified CT-guided coaxial needle technique with intracystic lidocaine pre-injection versus a conventional ultrasound-guided method with pigtail catheter drainage. By evaluating pain control during ethanol injection, procedural efficiency, safety profiles, and treatment outcomes between these two approaches, this study seeks to refine technical strategies for managing SHCs.

## Materials and methods

### Study design and population

Patients in this study were drawn from two centers: Center A (Dianjiang People’s Hospital of Chongqing) and B (Chongqing Traditional Chinese Medicine Hospital), which are located in different cities approximately 150 km apart. The study and procedures received approval from the Ethics Committees of both Dianjiang People’s Hospital of Chongqing and Chongqing Traditional Chinese Medicine Hospital (Ethics Approval Number: DYLL-KY-2019-027). This study was registered as a clinical trial (Registration ID: ChiCTR1900026082; 20/09/2019) and adhered to the Declaration of Helsinki guidelines. Written informed consent was obtained from all patients prior to participation. All procedures were performed in accordance with the relevant guidelines and regulations.

Patients with SHCs who underwent aspiration sclerotherapy between January 2019 and June 2023 were enrolled. All enrolled patients were diagnosed with SHCs using ultrasound or CT scan and met the following inclusion criteria: cyst diameter between 5 and 10 cm; symptoms of abdominal pain or discomfort; and reassurance due to increasing cyst size. Patients with previously treated cysts or lack of follow-up data were excluded.

### Treatment methods and follow-up

Both centers performed single-session procedures using 99.9% ethanol. All procedures were performed by two interventional radiologists at each center with more than 6 years (Center A) and 8 years (Center B) of experience in sclerotherapy procedures.

**Fig. 1:**
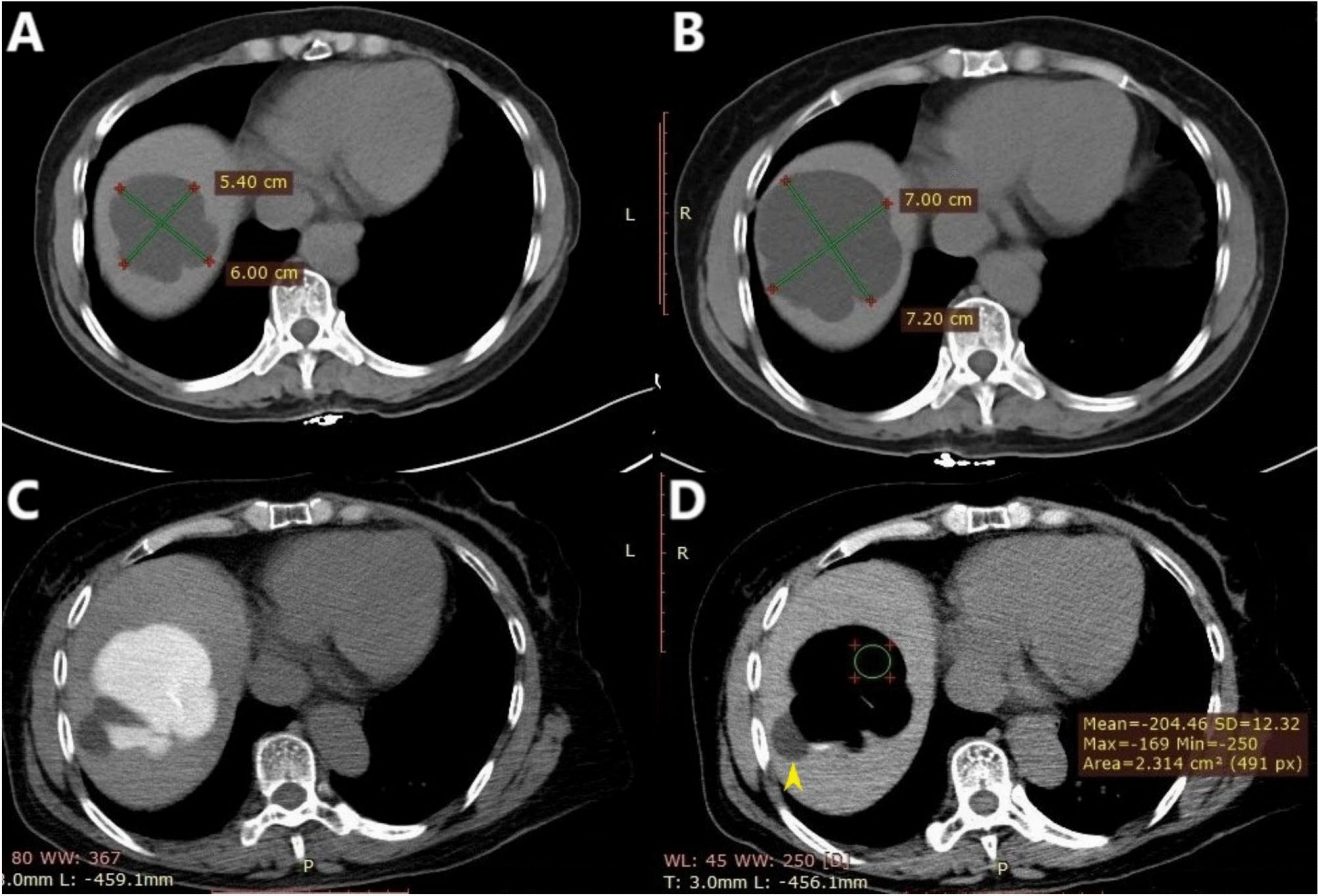
A 57-year-old woman presented with an enlarging hepatic cyst. The simple hepatic cyst (approximately 3–4 cm in size) was accidentally discovered in 2016 via ultrasound. CT performed in May 2018 and November 2019 revealed progressive enlargement of the cyst. The procedure was performed at Center A using the modified technique, and the patient’s prior (**A**,** B**) and interprocedural (**C**,** D**) CT images are shown below. (**A**) In May 2018, the cyst size was 5.4 cm × 6.0 cm × 3.6 cm. (**B**) In November 2019, the cyst size was 7.0 cm × 7.2 cm × 4.6 cm. Its volume increased from approximately 60 mL in May 2018 to 121 mL in November 2019. (**C**) In December 2019, the patient underwent percutaneous drainage. The needle tip was inserted close to the deep cyst wall, after which the contrast agent was injected. (**D**) The CT value of the intracystic fluid was − 204 HU, suggesting that the ethanol concentration exceeded 90%. A small cyst (yellow arrowhead) adjoining the large cyst was not treated.

**Fig. 2:**
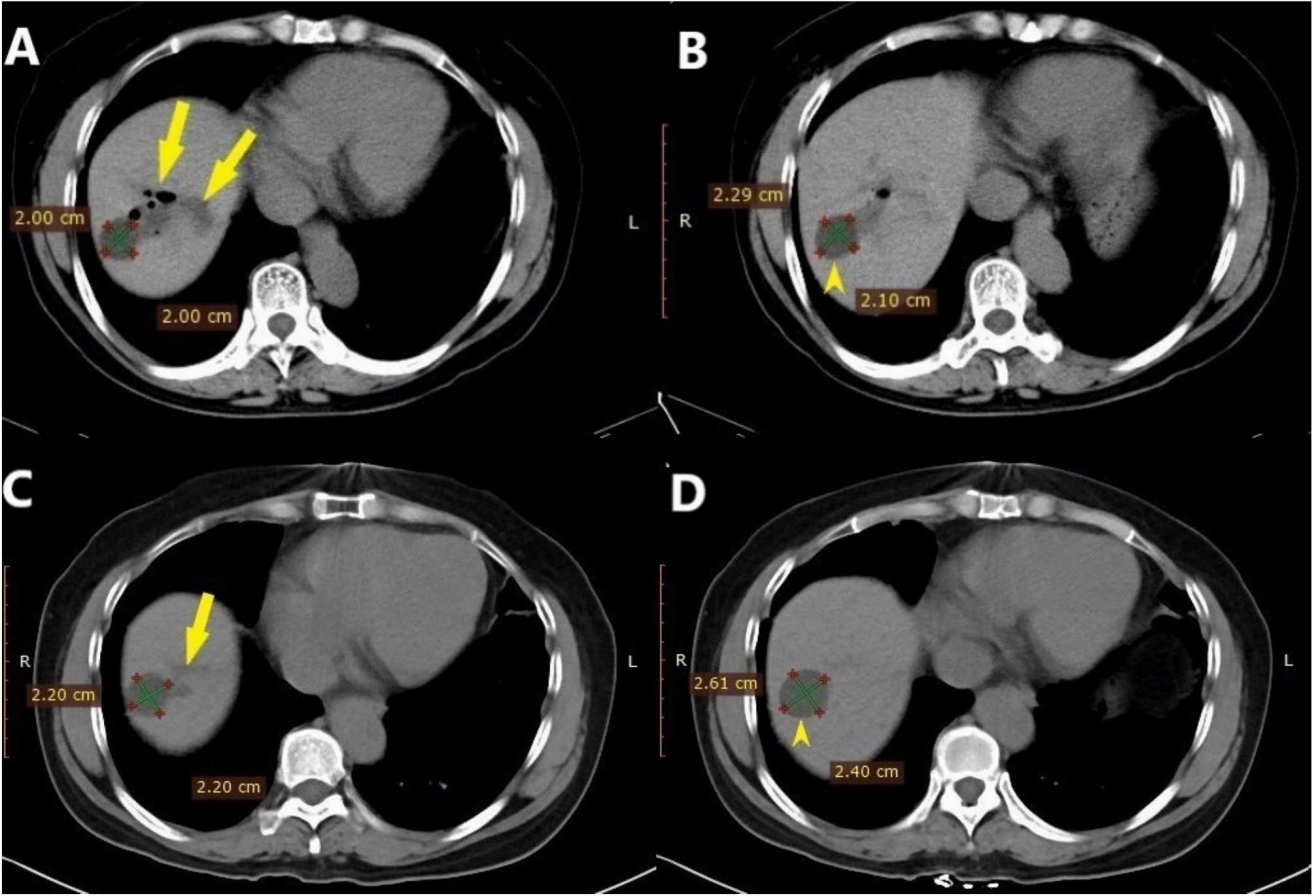
The patient did not complain of pain during injecting ethanol. The patient was discharged after 3 days without complications. Comparison images of immediate and 6-month postprocedural follow-ups. (**A**, **B**) In December 2019, CT performed immediately after the procedure revealed that the treated large cyst was significantly reduced in size with a small amount of gas remaining (yellow arrow) on consecutive scanning levels. The size of the untreated small cyst was 2.3 cm × 2.1 cm (yellow arrowhead). (**C**, **D**) In June 2020, CT performed after 6 months demonstrated that the treated large cyst had completely regressed with only the collapsed cyst wall remaining, and the untreated small cyst (2.6 cm × 2.4 cm) was slightly enlarged.

For Center A (modified technique), the procedure was performed using local anesthesia. A 20-gauge coaxial needle (Monopty 2016B Bard, Tempe, AZ, USA) was introduced into the cyst with computed tomography (CT) guidance. The stylet was subsequently removed, and a three-way drainage tube was attached. The needle tip was positioned beyond two-thirds of the cyst, taking care to prevent the needle from passively shifting out of the cyst during cyst wall collapse due to fluid volume decrease. A contrast agent was injected to confirm the lack of communication between the cyst and biliary system, and no contrast agent leakage was observed. Five milliliters of lidocaine were pre-injected into the cyst to control the distending pain caused by the subsequent ethanol injection. According to the formula for estimating the cyst volume (V = length × width × height × π/6), most of the cyst fluid was aspirated, leaving approximately 20 mL. Ethanol was then injected until the intracystic fluid was approximately 75% of the original cyst volume (not more than 300 mL). Generally, following the completion of the second or third repetitive cycle of aspiration and injection, the CT value of the cyst fluid will be less than − 190 HU (Fig. 1A–D), which signals the end of the replacement because the ethanol concentration within the cyst exceeds 90%. The patient’s original position was maintained for 5 min, and then the treatment was finished with ethanol withdrawal (Fig. 2A–D).

**Fig. 3:**
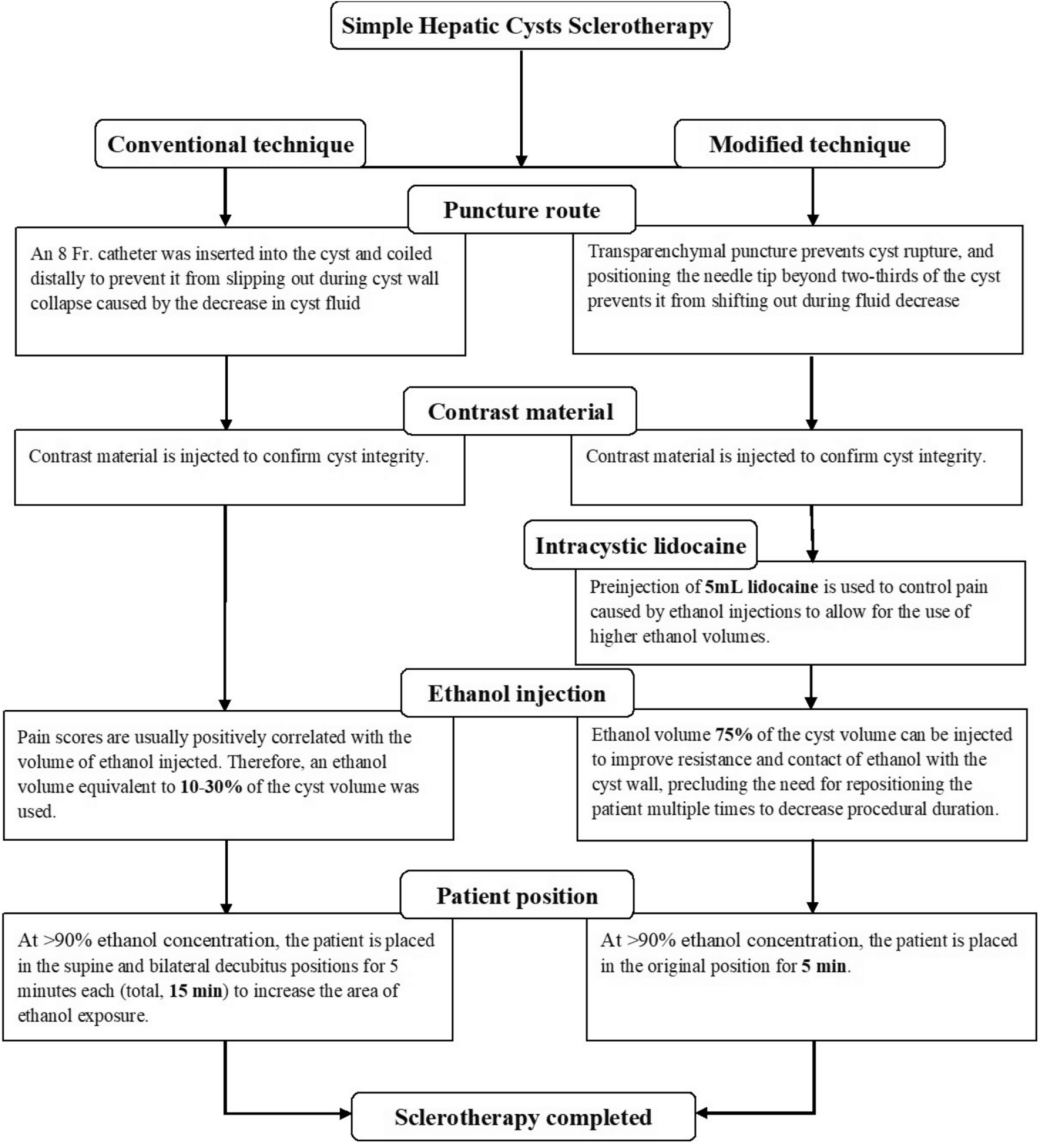
Flow diagram of comparison of the conventional and modified techniques.

For Center B (conventional technique), the standardized ultrasonography (US) guided cyst drainage procedure was performed. The cystic fluid was one-time aspirated as completely as possible using an 8-French pigtail catheter (Cook Medical, Bloomington, IN, USA). The amount of ethanol used was determined by the cyst’s size (equivalent to 10–30% of the volume of the aspirated fluid) and the patient’s pain response. The patients were placed in supine and bilateral decubitus positions for 5 min each, and then the ethanol was withdrawn. Following the completion of the single-session procedure, the pigtail catheter was immediately removed with no extended indwelling period. The flow diagram comparing the conventional and modified techniques is shown in Fig. 3.

No additional systemic analgesia or sedation was administered at either center beyond the local anesthesia at the puncture site. At Center A, 5 ml of 2% lidocaine were pre-injected into the cyst as described, while at Center B, no intracystic anesthetic was utilized. No patients required procedure termination due to pain at either center, although the total ethanol volume administered at Center B was sometimes limited by patient discomfort.

Patients in both groups visited the outpatient clinic 6 months after the procedure, and CT or US was performed to assess the volume of the treated cyst.

### Study endpoints

The primary endpoints in this study were pain during the injection of ethanol, procedural duration, and safety compared between the treatment strategies of the two groups. Secondary endpoints were cyst size reduction and symptomatic change.

### Data collection and analysis

An electronic patient database search was performed. Data for patients with SHCs who underwent aspiration sclerotherapy between January 2019 and June 2023 were accessed from electronic medical records. A dataset including baseline and all endpoints was assembled, which comprised basic patient information, clinical symptoms, ethanol dosage, pain during ethanol injection, procedure duration, complications, and initial and follow-up cyst volumes. For comparison, the cyst volume was rounded to the nearest tenth. Patients’ pain scores during ethanol injection were determined using the Visual Analog Scale (VAS; range, 0–10), categorized as no pain (0), mild (1–3), moderate (4–6), severe (7–9), and unbearable pain (10). A reduction in cyst size of 50%–80% was considered partial/complete regression. Symptomatic changes were classified into three categories: no, partial, or complete reduction of symptoms.

Statistical analysis was performed using SPSS version 26.0 (SPSS Inc., Chicago, IL, USA). The independent sample Student’s test was used to compare ethanol-related pain, ethanol dosage, and procedure duration. The Mann–Whitney U test was used to compare initial and follow-up cyst volumes. Categorical variables were analyzed using Pearson χ2 or Fisher’s exact test, as appropriate. A *p*-value of < 0.05 was considered statistically significant, and all *p* values are two-tailed.

## Result

**Fig. 4:**
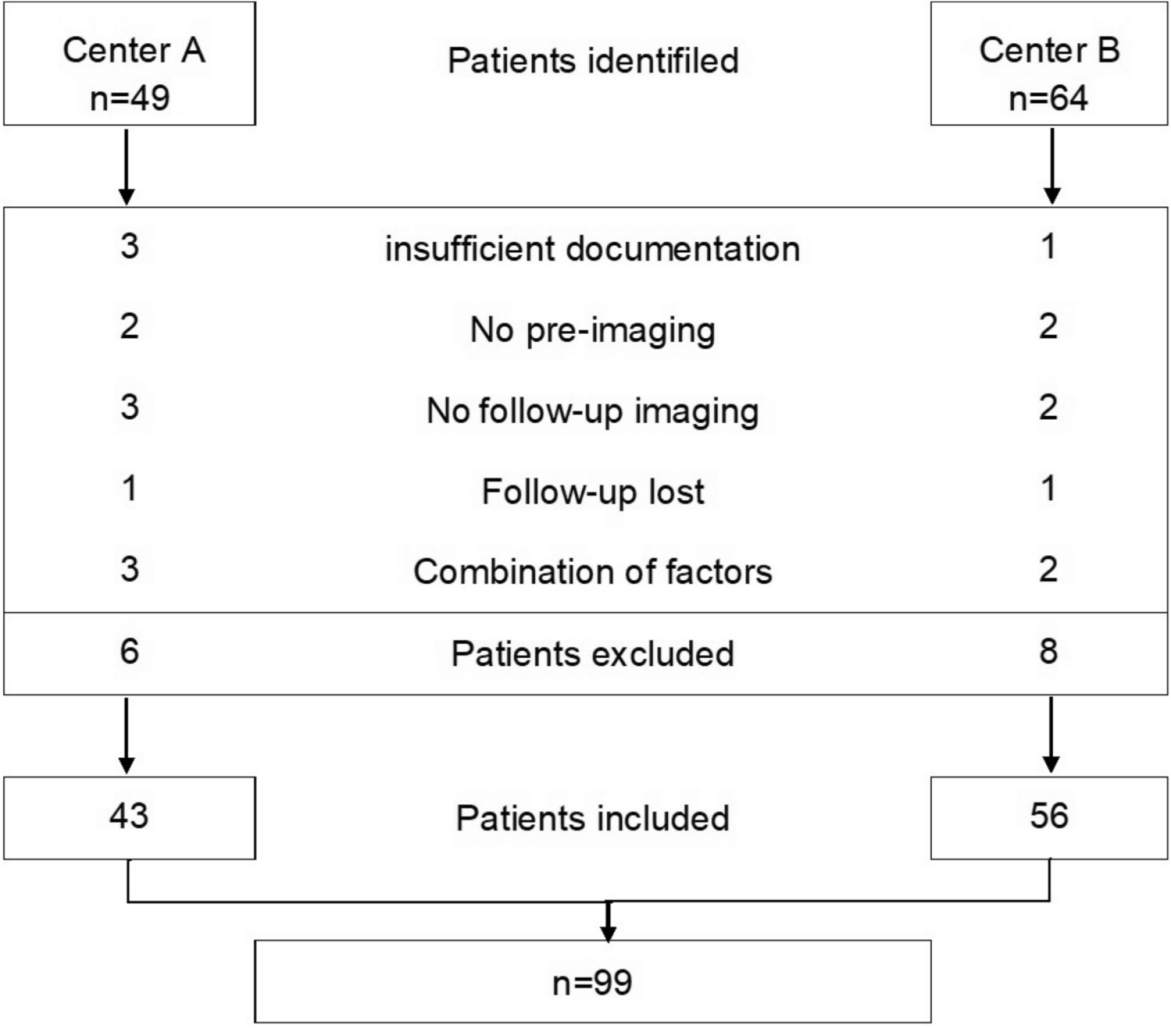
Flowchart: identification and selection of patients in Centers A and B.

A total of 113 patients were identified in Center A (n = 49) and Center B (n = 64). Of these, 99 patients from Center A (n = 43) and Center B (n = 56) were eligible for inclusion. Patients with insufficient documentation (n = 4), no pre-imaging (n = 4), or no follow-up imaging (n = 5) were excluded (Fig. 4).

The demographic and SHCs characteristics of the patients are summarized in Table 1.

**Table 1:** Demographic and SHCs characteristics.

Characteristics		Center A (*n* = 43)	Center B (*n* = 56)	*P* value
Age (years)		64 (60–71)	64 (60–73)	0.473
Sex	Male/Female	4(9.3)/39 (90.7)	9(16.1)/47 (83.9)	0.752
Laterality	Left/Right	13(30.2)/30 (69.8)	15 (26.8)/41 (73.2)	0.823
Clinical presentation				0.289
Abdominal malaise		8(18.6)	19(33.9)	
Abdominal pain		6(14.0)	7(12.5)	
Gradual enlarge		13(30.2)	17(30.4)	
Anxiety		16(37.2)	13(23.2)	
Cyst estimated volume (mL)		150 (120–185)	143 (121–180)	0.430

Values are median with interquartile range in parentheses, or number with percentage in parentheses.

Relevant treatment parameters are shown in Table 2.

**Table 2:** Relevant parameters of the treatment.

Parameter	Center A (*n* = 43)	Center B (*n* = 56)	*P* value
Puncture depth (cm)	7.0 ± 1.1	6.9 ± 1.0	0.829
Puncture angle (°)	9 (3.6–26)	18 (6.3–39.3)	0.148
Ethanol exposure duration (min)	11.2 ± 1.6	20.5 ± 3.4	< 0.001
Procedure duration (min)	29.9 ± 4.3	40.6 ± 4.2	< 0.001
VAS	1.2 ± 1.2	5.1 ± 1.1	< 0.001
Complication	0 (0)	4 (3.6)	0.437*
Alcohol toxicity	0	2	
Intracavitary hemorrhage	0	2	
Clinical symptomatic remission	38(88.4)	47(83.9)	0.575*
Abdominal malaise	6	15	
Abdominal pain	3	2	
Gradual enlarge	13	17	
Anxiety	16	13	
Therapeutic evaluation			0.728*
Complete regression rate	40(93.0)	50(89.3)	
Partial regression rate	3(7.0)	6(10.7)	
Follow-up cyst volume (mL)	2 (0–9.5)	5.5 (0–14.8)	0.287

Values are mean ± standard deviation, median with interquartile range in parentheses, or number with percentage in parentheses. The asterisk (*) means that Fisher’s exact test was performed.

**Fig. 5:**
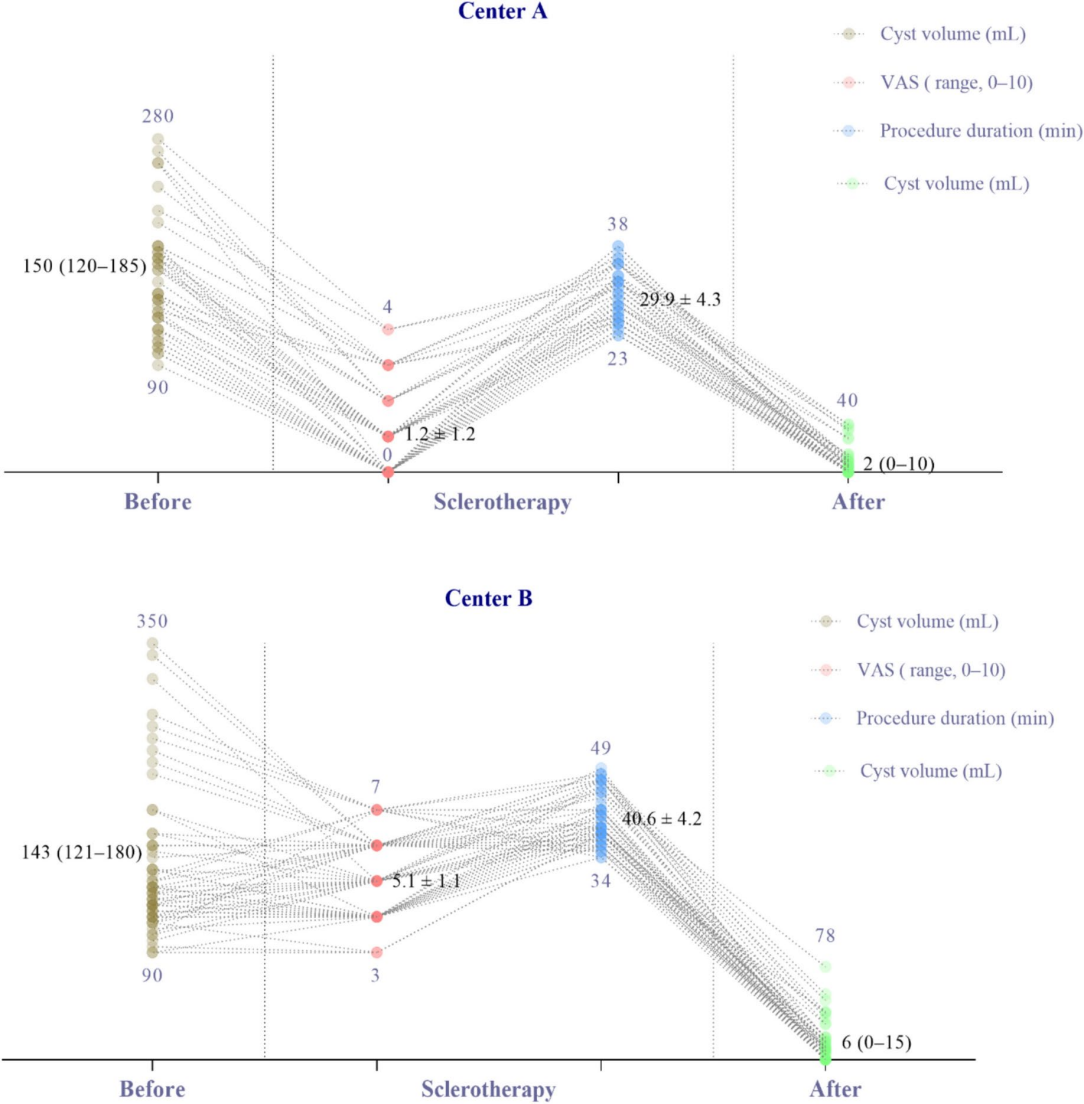
Comparison of cyst volumes before and 6 months after sclerotherapy, as well as VAS scores and procedure durations, between the two centers. To facilitate observation, VAS scores and procedure durations were magnified by 30 and 5 times, respectively, in the figure.

A small number of patients in Center A reported mild distention without significant pain, whereas all patients in Center B complained of more pronounced distention or pain during ethanol injection. The median VAS in Center B (5.1 ± 1.1) was significantly higher (*p* < 0.01) than in Center A (1.2 ± 1.2). Ethanol exposure and procedural durations were also higher (*p* < 0.01) in Center B (20.5 ± 3.4 min and 40.6 ± 4.2 min, respectively) compared to Center A (11.2 ± 1.6 min and 29.9 ± 4.3 min, respectively). Safety comparisons between the treatment strategies showed no significant differences, and no complications were observed in Center A (Fig. 5). The 6-month follow-up demonstrated similar success rates (partial and complete regression) and symptom relief between the two centers.

## Discussion

To the best of our knowledge, this is the first study to report on single-needle, single-session SHCs sclerotherapy incorporating intracystic lidocaine pre-injection for pain control to improve patient compliance and procedural efficiency. The modified technique demonstrates several key features and potential benefits when compared with the conventional ultrasound-guided percutaneous catheter drainage technique.

Single-session sclerotherapy eliminates patient discomfort caused by pigtail catheter indwelling and postprocedural management of the drain. The 20-gauge coaxial needle is less invasive to the body than the 8-French pigtail catheter^2,5^, and it has lower medical consumable costs for patients. The use of a three-way drainage tube prevents air from entering the cyst and facilitates ethanol exposure. However, for large cysts (diameter > 10 cm), traditional catheter drainage allows for repeated ethanol injections through the indwelling catheter post-procedure to ensure the efficacy of sclerotherapy, which is not possible with single-session sclerotherapy.

Before cyst aspiration, pre-injection of intracystic lidocaine, an inexpensive local anesthetic, provides convenient and quick control of pain, the most common adverse reaction encountered during ethanol sclerotherapy^4–6^. It allows lidocaine to contact a larger surface area of the cyst wall, as the cyst is still in its fully expanded state. This maximizes the anesthetic coverage of the cyst lining. Additionally, during the process of aspirating the cystic fluid and gradually reducing the fluid volume, the intracystic lidocaine can still maintain some contact area and duration with the cyst wall. This achieves better patient compliance and allows a larger ethanol volume to be injected, while avoiding more complex anesthesia methods such as conscious sedation, analgesics, or general anesthesia that would typically require an anesthesiologist’s presence and additional monitoring, as well as increased medical costs associated with them^7,8^.

In the conventional approach (Center B), ethanol injection immediately caused patient pain (higher VAS scores), which forced physicians to reduce the volume of ethanol injected to maintain patient tolerance. This reduction in ethanol volume meant that longer exposure times were needed to achieve the desired therapeutic effect, thus extending the entire procedure duration. Conversely, prolonged exposure time could further exacerbate patient discomfort, creating a cycle that potentially limited treatment efficacy. In contrast, the lidocaine pre-injection in the modified technique (Center A) disrupted this cycle by significantly reducing pain during ethanol injection (lower VAS scores), allowing for substantially larger ethanol volumes (75% versus 10–30% of cyst volume), which not only increased the contact area between ethanol and the cyst wall but also reduced the time needed to reach effective concentrations, and eliminated the need for additional patient repositioning in bilateral decubitus positions, thereby shortening overall exposure time and procedure duration. This bidirectional relationship allows a single technical modification—intracystic lidocaine pre-injection—to simultaneously improve multiple procedural parameters.

The cystic fluid was not one-time completely aspirated in the modified technique. Therefore, 1–2 mL of cyst fluid was allowed to be steadily aspirated without experiencing negative pressure before each injection to confirm that the needle tip is within the cyst, ensuring that ethanol is injected safely and avoiding the injection of any air present in the drainage tube into the cyst. Additionally, multiple aspirations can clean out coagulated necrotic protein fragments, facilitating ethanol exposure. High-concentration ethanol can lyse fluid-producing endothelial cells in 3 min, leading to the regression of the cyst^9,10^. Consequently, an exposure duration of 5 min with an ethanol concentration exceeding 90% can achieve efficacy and reduce the influencing factors of ethanol intoxication^6,11^.

Ultrasound guidance is radiation-free and provides real-time imaging of cysts, helping operators accurately locate and monitor the treatment process. CT guidance, on the other hand, offers higher resolution, clearer three-dimensional images, and the ability to monitor ethanol concentration compared to ultrasound. The concentration of intracystic ethanol can be monitored by the CT value based on the radiation principle of different X-ray absorption coefficients of substances with different densities or concentrations. The curative effect is better when the ethanol concentration exceeds 90%, corresponding to the CT value of ethanol in the cyst is less than − 190 HU^9,12^. Simultaneously, using the accurate and objective CT value instead of the original visual subjective determination of ethanol concentration as a signal to stop using ethanol could make the procedure more standardized and the curative effect more stable^9,12^.

Despite these promising advantages of the modified technique, several limitations of this study should be acknowledged. First, large cysts (exceeding 10 cm in diameter) were not included in the modified technique due to concerns about efficacy and safety. Additionally, the modified technique did not encounter SHCs larger than 300 mL in volume. Second, the postoperative follow-up of patients utilized both CT and US examination techniques, which is not uniform. Third, while anxiety was listed as a common indication for treatment, it was not quantified using standardized psychological assessment tools, relying instead on qualitative documentation in medical records. Fourth, the clinical appropriateness of interventional procedures was not carefully evaluated. For patients whose primary symptom is anxiety, non-interventional approaches such as patient education and psychological support are generally more suitable first-line strategies. Fifth, the sample size is small, which may obscure defects.

## Conclusion

In conclusion, this study described and assessed a CT-guided percutaneous single-session ethanol sclerotherapy technique that offers a painless, efficient, safe, effective, inexpensive, and minimally invasive alternative for SHCs less than 300 mL in estimated volume.

## Data Availability

The data used to support the findings of this study are included within the article.
